# The Treatment of Typhoid Fever

**Published:** 1889-10

**Authors:** 


### THE TREATMENT OF TYPHOID FEVER.

Many interesting papers have been written recently on this ever entertaining subject, and much has been said that adds greatly to our knowledge of the pathology and treatment of typhoid fever. The consensus of the best medical opinion tends surely away from the dread of hyperpyrexia *per se*, and against the use of antipyretics as the *sine qua non* in the treatment. The results obtained with the coal tar series of antipyretics are certainly discouraging. To control the temperature with any of them they must be administered in constantly increasing doses. They increase the tendency to heart failure, and often produce fatal collapse. The duration of the disease is not shortened by their use; on the contrary, statistics seem to show that it is prolonged. The elimination of effete material is apparently interferred with. The death rate, where they have been employed systematically, is exceedingly high, showing, in New York City, where they have, perhaps, been used more extensively than anywhere else, an average mortality of about 21.9 per cent. In 18,612 cases under different methods of treatment collected by Murchison, the rate of mortality was 18.62 per cent. Contrast the above figures with those obtained by Brand under the cold water treatment:

Cold bathing fairly strict, 1879–1889, 5,573 cases, mortality 3.9 per cent; cold bathing very strict, 1,223 cases, mortality 1 per cent. In 2,150 cases collected by Brand from various sources, under "strict cold baths," before the fifth day there was not a single death. A careful consideration of the above statistics must certainly set us thinking. In the light of these facts is not our duty made plain?

Dr. Frederick C. Shattuck, of Boston, in a paper published in the *Boston Medical and Surgical Journal*, September 5th, says of the use of antipyretics in typhoid fever:

"The internal antipyretics I used a good deal in 1886, in primary attacks; scarcely at all in 1887, and in 1888 only on the rarest occasions in relapses. I now give them only in the comparatively rare cases where the fever itself seems to interfere with the comfort of the patient." He says further: "Whether we err here or not, that pyrexia is not the evil spirit we thought it to be, seems to be, for instance, clearly shown by Glasser's analysis of 200 fatal cases of typhoid out of 3.000 treated in the Hamburg General Hospital. In only eight per cent. of the fatal cases was the average temperature higher than that of the ordinary mild type of the disease, and in none of the fatal cases were higher points reached than we all see in cases which terminate favorably."

Brand and his followers attribute the beneficial effects of cold baths to the stimulation of the nervous centers, preventing pulmonary complications, rather than to the reduction of temperature.

In the few cases seen late in the disease where cold baths are not admissible, tepid baths, long continued, act promptly in reducing the temperature, and are very soothing to the patient.

The antiseptic treatment, with calomel purges, has not yielded as good results as were so confidently expected of it.

Alcohol must continue to be one of our most valuable therapeutic agents in those cases where it is indicated. Its rapid oxidation prevents the oxidation of the tissues themselves, and the consequent wasting.

Judging by the clinical results, one must admit that Brand's cold water treatment offers the greatest hope to the patient. Where the surroundings of patients are such that his plan of treatment cannot be carried out successfully, we must rely upon expectant treatment, with diet, tepid baths, etc., as indicated. We wish to put ourselves on record against the use of the internal antipyretics as a routine treatment.

With the present lights before us, the failure to properly disinfect the excreta of patients suffering with typhoid fever is almost criminal, and the physician who fails to do his duty in this respect, will assuredly, ultimately, be held responsible.

